# Evaluation of a visual acuity eHealth tool in patients with cataract

**DOI:** 10.1097/j.jcrs.0000000000001108

**Published:** 2022-12-06

**Authors:** Joukje C. Wanten, Noël J.C. Bauer, Janneau L.J. Claessens, Thomas van Amelsfort, Tos T.J.M. Berendschot, Robert P.L. Wisse, Rudy M.M.A. Nuijts

**Affiliations:** From the University Eye Clinic Maastricht, Maastricht University Medical Center+, Maastricht, the Netherlands (Wanten, Bauer, van Amelsfort, Berendschot, Nuijts); Department of Ophthalmology, University Medical Center Utrecht, Utrecht, the Netherlands (Claessens, Wisse).

## Abstract

To validate the Easee web-based tool for the assessment of visual acuity in patients who underwent cataract surgery.

**Setting:**

University Eye Clinic Maastricht, Maastricht, the Netherlands.

**Design:**

Prospective method comparison study.

**Methods:**

Subjects aged between 18 and 69 years who underwent cataract surgery on 1 or both eyes at the Maastricht University Medical Center+ were eligible to participate in this study. The uncorrected (UDVA) and corrected distance visual acuity (CDVA) assessments were performed using the web-based tool (index test) and conventional ETDRS and Snellen charts (reference tests). The outcomes of the different tests were expressed in logMAR, and a difference of <0.15 logMAR was considered clinically acceptable.

**Results:**

46 subjects with 75 operated eyes were included in this study. The difference of the UDVA between the web-based tool and ETDRS or Snellen was −0.05 ± 0.10 logMAR (*P* < .001 [0.15; −0.26]) and −0.04 ± 0.15 logMAR (*P* = .018 [0.24; −0.33]), respectively. For the CDVA, these differences were −0.04 ± 0.08 logMAR (*P* < .001 [0.13; −0.21]) and −0.07 ± 0.10 logMAR (*P* < .001 [0.13; −0.27]), respectively. The Pearson correlation coefficients between the web-based tool and ETDRS were maximally 0.94 and compared with Snellen 0.92. In total, 73% to 88% of the visual acuity measurement differences were within 0.15 logMAR.

**Conclusions:**

The web-based tool was validated for the assessment of visual acuity in patients who underwent cataract surgery and showed clinically acceptable outcomes in up to 88% of patients. Most of the participants had a positive attitude toward the web-based tool, which requires basic digital skills.

## Abstract 2

The Easee eHealth tool was validated for the assessment of visual acuity in patients who underwent cataract surgery and showed clinically acceptable outcomes in up to 88% of patients.

Cataract is the world's leading cause of age-related vision loss.^1^ During the past few decades, it has become one of the most performed surgeries worldwide and the number of procedures is likely to increase.^2^ The corresponding postoperative care includes frequent and rather time-consuming routine check-up appointments. In combination with the low incidence of serious sight-threatening complications, optimizing the postoperative cataract care pathway through eHealth technology is a logical next step in improving the patient journey.

The efficiency of the postoperative care could be enhanced by using remote care using teleconsultation and (online) remote measurements. Over the past few years, organizing remote care has accelerated, partly because of the COVID-19 pandemic.^3^ Several clinics have already implemented this kind of care and replaced 1 or more regular clinical follow-up examinations by telephone consultations.^4^ However, these teleconsultations are only partly applicable in ophthalmologic care because they lack objective outcome parameters for visual acuity and refractive state. Upcoming eHealth applications which provide the opportunity of self-monitoring and collecting objective outcome parameters may offer a solution. Utilization of these applications will lower the burden on patients after cataract surgery by saving follow-up visits at the outpatient clinic, which may improve efficiency and lower costs. The increased use of digital tools in general supports the implementation of eHealth solutions.

One of these eHealth applications is the Easee web-based tool that allows patients to individually assess their visual acuity and corresponding refraction using a smartphone and computer screen. Recently, noninferiority was shown for refraction measurements of this tool compared with manifest refraction obtained from standard measurements at the outpatient clinic in a healthy study population. Besides, the web-based tool and ETDRS chart showed similar results for the uncorrected distance visual acuity (UDVA) with mean values of 0.33 ± 0.30 logMAR and 0.39 ± 0.39 logMAR (*P* = .21), respectively.^5^

The aim of this study was to validate the web-based tool for assessment of the visual acuity in patients who underwent cataract surgery. We hypothesize agreement between the visual acuity measurements performed by the web-based tool as compared with the conventional assessments.

## METHODS

### Test–Retest

Firstly, a test–retest analysis was performed among 5 healthy volunteers by measuring the right eye UDVA using the Snellen, ETDRS visual acuity charts, and the web-based tool. The measurements were performed at 3 different dates by the same individual under the same controlled and optimized circumstances, providing an indication of intraindividual variability of these tests.

### Study Design and Recruitment

From November 2020 to March 2021, a total of 46 participants were recruited from the University Eye Clinic of Maastricht University Medical Center (MUMC+). Subjects were eligible if they were aged between 18 and 69 years, underwent cataract surgery on 1 or both eyes, and were able to perform the web-based tool in Dutch, German, or English. The age limit of 69 years was selected based on the data of European statistics concerning digital skills to minimize the effects of digital proficiency on study outcomes.^6^ All participants were informed about the study in advance and signed an informed consent before enrolment. This hospital-based validation study was approved by the local medical ethics committee and institutional review board of the MUMC+ (Maastricht, the Netherlands). The study was executed in accordance with the tenets of the Declaration of Helsinki.

### Conventional (Reference Tests) and Web-Based (Index Test) Assessments

Both UDVA and corrected distance visual acuity (CDVA) were assessed using the Snellen and ETDRS charts as reference tests. The Snellen chart was routinely assessed by an optometrist at the postoperative visit before study enrolment. For the Snellen chart, the line assessment method was used. After study enrolment, visual acuity was assessed using the ETDRS chart by the researcher. The chart was placed 4 m from the subject, and the last attempted line on the ETDRS chart was determined until no optotypes could be distinguished. The total number of correctly identified optotypes was added to the score of the last attempted line to determine a logMAR score. Monocular CDVA measurements were performed using trial frames with the subjective manifest refraction as routinely measured by the optometrist.

**Figure 1.**
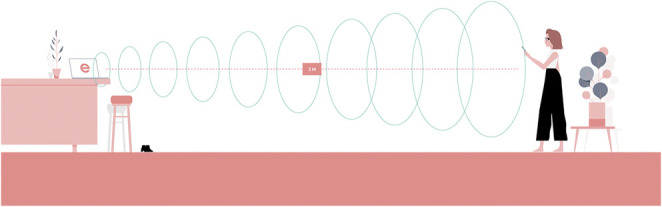
The web-based tool is performed by the patient by using a computer, 3 m distance, and a smartphone used as a remote controller.

The web-based tool (Easee B.V.) is an online visual function test using a computer screen and a smartphone (Figure 1). The smartphone is used as a remote controller to submit the input of the user from a distance of 3 m from the computer screen. All participants performed the web-based tool at the outpatient clinic after their (regular) postoperative visit, under controlled and optimized conditions, using a commonly used smartphone (Samsung Galaxy S6) and a laptop (Dell Latitude 5501, 15.6 inch). The test consisted of 3 parts with audio and visually instructed guidance: intake, arrangement of the test (calibrating and connecting the screen, placing a chair at 3 m distance), and performing the test. A short demo video illustrated the purpose of the web-based tool. During the test, a sequence of optotypes (tumbling-E and proprietary optotypes) was displayed that had to be identified correctly by the subject. The application used built-in algorithms to check the consistency of the input.

All participants performed the web-based test twice. Firstly, monocular UDVA measurements were performed and secondly monocular CDVA measurements. CDVA was assessed using trial frames with the subjective manifest refraction as used during the reference tests. Subjects could get assistance in using the smartphone and were reminded to cover up the appropriate eye during the tests. The amount of time the participants needed to perform the web-based tool was collected. Online refractive measurements were not performed in this study.

All assessments were performed using a fixed sequence: All operated eyes were routinely examined performing the Snellen UDVA and CDVA, first left and then the right eye. Subsequently, the researcher conducted the ETDRS measurements followed by measurements using the web-based tool in the same abovementioned sequence. All participants were unaware of their results.

### Questionnaires

An exploratory questionnaire was performed to assess pretest and posttest confidence of subjects toward the web-based tool. Questions pertained to the recommendation of the web-based tool to other patients, to the level of confidence of the subjects in their results, and to the amount of assistance during the tests. Outcomes were scored for every individual subject using a Likert scale (ranging from “strongly agree” to “strongly disagree”). In addition, the *digital skills indicator survey*, derived from the Eurostat survey on ICT usage by individuals, was performed.^7^

### Sample Size

The sample size calculation of 46 participants was based on a desired limit of agreement (LoA) of 0.01 logMAR and an assumed SD of 0.02 logMAR.^8^

### Statistical Analysis

Analyses were performed using SPSS (v. 25, IBM Corp.). An outcome was considered statistically significant when the *P* value was ≤0.05. Frequencies and descriptive statistics were used to summarize the baseline criteria and analyze the distribution of the variables. Left and right eyes were analyzed both separately and combined.

When using the ETDRS and web-based tool, UDVA and CDVA were expressed in logMAR. Each optotype of the ETDRS chart had a score of 0.02 log units, and 1 line represented 0.10 logMAR. The Snellen test was measured in decimals and afterward converted to logMAR. The analysis of variance between the web-based tool, Snellen, and ETDRS charts was performed using general linear model–repeated measures. Differences between visual acuity outcomes of the individual tests were compared using paired *t* tests. Data were displayed in scatterplots, and the related Pearson correlation coefficients were calculated. Bland Altman plots were used to visualize the agreement between the web-based tool and the reference tests.^9^ A difference ≥0.15 logMAR was considered clinically relevant because this is the usual intraindividual variability in repeated visual acuity measurements.^10–12^

## RESULTS

Firstly, a small test–retest sample was performed among 5 healthy volunteers and showed SDs for the ETDRS of 0.05 logMAR, Snellen 0.04 logMAR, and web-based tool of 0.08 logMAR. The study population consisted of 22 women (48%) and 24 men (52%) with a mean age of 62.8 ± 7.1 years (ranging from 26 to 69 years). Bilateral cataract surgery was performed in 29 patients (63%). The manifest refraction spherical equivalent (MRSE) was −0.41 ± 0.84 diopters (D) for 41 operated right eyes and −0.64 ± 1.33 D for 34 operated left eyes. Most of the 44 patients (96%) had basic digital skills.

A total of 75 operated eyes completed the assessments using the web-based tool and the conventional ETDRS chart. Outcomes of the web-based tool, Snellen, and ETDRS chart showed a significant visual acuity underestimation of the index test, when compared with the reference tests for right UDVA and CDVA, and left CDVA (Table 1). The differences between the visual acuity outcomes of the web-based tool were maximally −0.07 ± 0.10 logMAR (*P* < .001) compared with the ETDRS chart and had a maximal value of −0.08 ± 0.12 logMAR (*P* < .001) compared with the Snellen chart. The correlation ranged from 0.70 to 0.94 and up to 88.2% of the web-based outcomes was within the clinically significant difference cutoff value of ±0.15 logMAR (Table 2).

**Table 1.** Outcomes of different visual acuity assessments

Parameter	Visual acuity test	N	Mean ± SD (logMAR)	Variance *P* value
UDVA RE	ETDRS Snellen Web-based	41 39 41	0.15 ± 0.30 0.17 ± 0.38 0.22 ± 0.30	.001
UDVA LE	ETDRS Snellen Web-based	34 32 34	0.14 ± 0.27 0.11 ± 0.23 0.17 ± 0.27	.060
CDVA RE	ETDRS Snellen Web-based	41 37 41	0.03 ± 0.22 −0.04 ± 0.12 0.06 ± 0.21	.002
CDVA LE	ETDRS Snellen Web-based	34 33 34	−0.03 ± 0.15 −0.05 ± 0.08 0.02 ± 0.14	<.001

RE = right eye; LE = left eye.

Mean UDVA and CDVA scores of the right and left eyes in logMAR (±SD). The variance (*P* values) between the web-based tool, Snellen, and ETDRS charts was assessed using general linear model–repeated measures.

**Table 2.** Comparison of visual acuity outcomes of different tests

Parameter	Difference between tests	Mean ± SD (logMAR)	*P* value	Pearson correlation^a^	% within ±0.15 logMAR
UDVA RE	ETDRS: web-based Snellen: web-based ETDRS: Snellen	−0.07 ± 0.10 −0.04 ± 0.16 −0.03 ± 0.13	<.001 .125 .172	0.94 0.92 0.95	82.9 76.9 76.9
UDVA LE	ETDRS: web-based Snellen: web-based ETDRS: Snellen	−0.04 ± 0.10 −0.04 ± 0.13 0.00 ± 0.11	.037 .060 .925	0.93 0.88 0.90	88.2 75.0 87.5
CDVA RE	ETDRS: web-based Snellen: web-based ETDRS: Snellen	−0.03 ± 0.09 −0.08 ± 0.12 0.04 ± 0.09	.029 <.001 .013	0.91 0.70 0.84	85.4 73.0 89.2
CDVA LE	ETDRS: web-based Snellen: web-based ETDRS: Snellen	−0.05 ± 0.08 −0.06 ± 0.08 0.01 ± 0.09	.001 <.001 .697	0.84 0.72 0.68	85.3 81.8 90.9

RE = right eye; LE = left eye.

Outcomes paired *t* tests and Pearson correlations between the different visual acuity tests

a All outcomes of the Pearson correlation coefficients had a *P* value of <0.001

**Figure 2.**
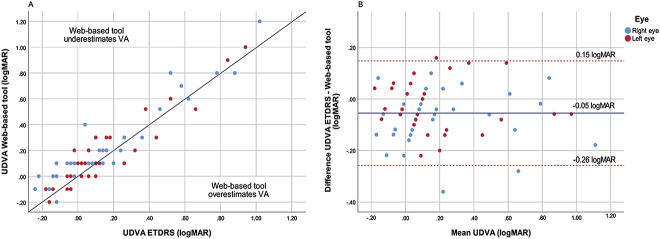
*A*: Scatterplot UDVA of the web-based tool and ETDRS chart for the right and left eyes. The *line* presents the line of equality. *B*: Bland-Altman plot of UDVA determined by the web-based and ETDRS chart. The *blue line* represents the mean value, and the *red dashed lines* represent the ±1.96 SD (95% limits of agreement).

**Figure 3.**
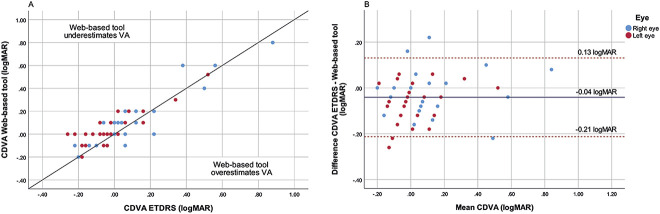
*A*: Scatterplot CDVA of the web-based tool and ETDRS chart for the right and left eyes. The *line* presents the line of equality. *B*: Bland-Altman plot of CDVA determined by the web-based and ETDRS chart. The *blue line* represents the mean value, and the *red dashed lines* represent the ±1.96 SD (95% limits of agreement).

The correlation coefficients between the web-based tool and ETDRS chart of both eyes combined for UDVA and CDVA were 0.94 and 0.89, respectively (both *P* < .001) (Figures 2, A and 3, A). The corresponding Bland Altman plots showed 95% LoAs ranging from 0.15 to −0.26 logMAR and 0.13 to −0.21 logMAR, respectively (Figures 2, B and 3, B). For the comparison between the scores of the web-based tool and Snellen chart, scatterplots and corresponding Bland Altman plots can be found in the Appendix (Supplemental Figures 1 and 2, http://links.lww.com/JRS/A761, http://links.lww.com/JRS/A762). The Snellen CDVA score had a statistically significant mean difference of maximally −0.08 ± 0.12 logMAR with the web-based outcomes. The UDVA and CDVA between Snellen and the web-based tool had a correlation coefficient of 0.89 and 0.71 (both *P* < .001), respectively. The 95% LoA ranged from 0.24 to −0.33 logMAR for the UDVA and from 0.13 to −0.27 logMAR for the CDVA.

**Figure 4.**
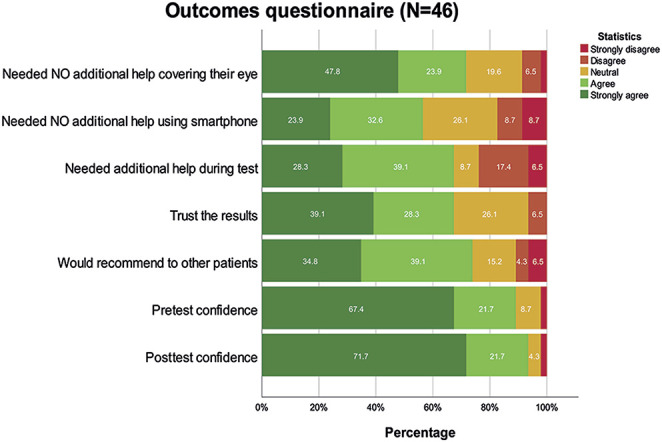
Outcomes of the questionnaire about the attitude and experiences of subjects using the web-based tool. Every questionnaire item was scored using a 5-level Likert scale. The outcomes are given in percentages.

The mean time for performing the web-based tool was for the UDVA of the first and second tested eye 98 ± 45 and 88 ± 48 seconds, respectively. The CDVA assessment was completed in 73 ± 43 seconds for the first and 65 ± 22 for the second tested eye. Questionnaire outcomes are shown in Figure 4. The distribution of these outcomes was skewed and therefore not suitable for additional analyses.

## DISCUSSION

The aim of this research was to validate the web-based tool for visual acuity assessment among patients who underwent cataract surgery. This study demonstrates statistically significant differences for both UDVA and CDVA scores between the web-based tool and the gold standard ETDRS chart of maximally −0.07 ± 0.10 logMAR and −0.05 ± 0.08 logMAR, respectively. Apparently, the web-based tool underestimates visual acuity, falling within the clinically acceptable cutoff of 0.15 logMAR. The Pearson correlation coefficients show a good reliability. However, it must be noted that this correlation cannot be defined as agreement because it only measures association.^13^ The Bland Altman plots show a wide distribution between these tests, with a 95% LoA maximum variation between 0.15 and −0.26 logMAR. However, up to 88% of the patients' visual acuity outcome differences were within the range of ±0.15 logMAR. Patients who were out of this range had either higher or worse visual acuity scores. Based on these results, we believe the web-based tool has an acceptable accuracy for clinical application.

Since the Snellen chart is the most commonly used test in daily clinical practice, the web-based outcomes were also compared with those obtained using a Snellen chart. Only the CDVA score showed a statistically significant mean difference of maximally −0.08 ± 0.12 logMAR. The Pearson correlation coefficients showed a reduced reliability compared with the correlation of the web-based tool and ETDRS chart. In total, 82% of the patients had a visual acuity outcome difference within ±0.15 logMAR. Furthermore, explorative analyses did not reveal any consistent or useful relationships between questionnaire results and visual acuity outcomes.

Questionnaire outcomes showed that most of the participants had a positive attitude toward the web-based tool. The net promotor score for the confidence toward the web-based outcomes was 86.9 before and 91.1 after performing the test. Generally, the amount of time the participants needed to perform the web-based assessment declined over the course of measurements. We observed a learning curve for completing the test in which the last performed measurement was completed the fastest.

A study using the web-based tool indicated this test as a valid and safe method for measuring visual acuity and refraction in healthy eyes. They found no difference between UDVA assessed by the web-based tool and an ETDRS chart, with mean values of 0.33 ± 0.30 and 0.39 ± 0.39 logMAR (*P* = .21), respectively.^5^ A study among patients with keratoconus showed an UDVA mean difference of −0.01 logMAR (*P* = .76), comparing ETDRS and the web-based tool, with a broad distribution including a LoA of −0.63 to 0.60 logMAR, albeit in subjects with a lower visual acuity.^14^

Several previous studies compared digital tools with conventional visual acuity charts, including the “Eye Chart,” “Peek Acuity,” and “Vision at Home” tool. These tools showed maximal mean differences of −0.01 logMAR (LoA of −0.21 to 0.19), 0.01 logMAR (LoA of −0.40 to 0.42), and 0.06 logMAR (LoA of −0.23 to 0.35) when compared with the ETDRS chart, respectively. The “Eye Chart” and “Peek Acuity” were tested among healthy adults, with a mean age of 64 and 65 years, respectively. The “Vision at Home” tool was performed by adolescents, adults, and elderly. Only the “Peek Acuity” tool was tested in the home and clinical environment, the other tools were tested in the controlled clinical environment. The tests were all performed using (habitual) spectacle correction. In comparison with the web-based tool, these visual acuity tests have an equivalent or better performance but were tested with a different methodology: The digital tests were all smartphone-based and were performed at different testing distances (the “Peek Acuity” and “Vision at Home” tool at 2 m testing distance and the “Eye Chart” at 4 feet [1.20 m] distance), and the visual acuities were scored using different methods (including letter-by-letter and line assignment) and not specifically assessed among patients who previously underwent cataract surgery.^15–17^ This might be an explanation for the discrepancies in outcomes compared with the web-based tool in this study. According to a systematic review, digital tools were in general less accurate in measuring visual acuity compared with conventional charts and showed wide distributions.^18^

There is consensus that outcomes of different visual acuity assessments vary.^11^ This variability is partly due to the psychophysical origin of the tests. Other reasons can be the design structure of the charts (decimal or logMAR), the optotypes used, the scoring methods, and the conditions under which the test is administered. Previous studies demonstrated that a decimal chart overestimates visual acuity compared with a logMAR chart.^19^ Concerning the scoring methods, the letter-by-letter method is more accurate compared with the line assignment method.^20^ For this study, the difference in scoring methods is presumably the primary factor that has caused some bias. The ETDRS chart is scored by the letter-by-letter method, the Snellen chart by the line assignment method, and the web-based tool by using an algorithm with a customized letter-by-letter method. Furthermore, the web-based tool has 7 optotypes in each line instead of 5. The abovementioned characteristics contribute to the general variability between visual acuity tests. If the postcataract pathway will represent a combination of both in-hospital and at home visual acuity tests, this should be taken into account. Nevertheless, a combination of these testing procedures can be very helpful because it offers flexibility for both patients and clinicians. Besides, the main aim of visual acuity testing after cataract surgery is a safety check for postoperative complications. In the case of a nonsignificant complication, an underestimation of the visual acuity up to 1.5 lines would be of lesser clinical relevance. Therefore, the variability of this visual acuity tool does not have a negative influence on the patient pathway but offers an additional screening opportunity.

In addition, the usage of different optotypes in assessments may affect the outcomes as well. Previous studies compared Landolt rings with numbers and showed higher visual acuity outcomes (0.13 ± 0.14 logMAR) using number optotypes.^21^ Other confirmed these lower outcomes when using Landolt rings compared with the Snellen (tumbling-)E chart or LEA symbols.^21^ In yet another study, there was no significant difference observed between visual acuity outcomes assessed by the Landolt and ETDRS chart among healthy and cataract eyes.^22^ These mentioned observations may have contributed to some discrepancies in our results. The web-based tool used tumbling-E optotypes, and the conventional charts had letter optotypes. Finally, the outcomes strongly depend on the achieved visual acuity of the tested subjects. Patients with the better scores tend to have more accurate visual acuity outcomes using the web-based tool.^5^ However, our outcomes did not confirm these findings, presumably due to the high overall visual acuity in our study population.

The limitation of this study was the fact that the web-based tool was only performed once. As a consequence, no test–retest or intraindividual consistency results were obtained from patients, which could have (partly) explained the variance between the gold standard and the web-based tool. Nevertheless, the web-based tool is considered to have a high test–retest reproducibility because of the nonvariable interpretation of patient responses by the tool.^5^ However, our additional test–retest analysis in healthy volunteers indicates that the variability of the web-based tool is up to twice as high compared with the conventional charts. Previous research showed a mean test–retest variability of the ETDRS and Snellen charts of 0.10 logMAR (LoA of −0.18 to 0.18) and −0.02 logMAR (LoA of −0.35 to 0.31), respectively.^12^ For assessing the intraindividual consistency among postcataract users of the web-based tool, further research is necessary. We can conclude that the differences among all 3 test outcomes in this study confirm the great variability generally observed.

Since the visual acuity assessments were performed under ideal conditions, it is expected that when patients perform the web-based tool in their home environment, outcomes may have both a lower reliability and greater variability. Other aspects, which may have influenced the outcomes, are the nonrandomized test sequence and duration of testing. The web-based tool was performed after the ETDRS chart assessment, which could have resulted in less accurate visual acuity outcomes using the web-based tool due to fatigue. In our results, no learning curve pattern could be demonstrated between first and second examined eyes. The subjects were blinded to the results to limit the performance bias. However, observation bias could not completely be prevented.

For implementation of a digital tool, practical issues must be taken into account. In this study, both UDVA and CDVA were evaluated. When performing an online visual acuity test in the home environment, the CDVA can only be measured after the patient has received his/her newly prescribed spectacles. Hence, directly after cataract surgery, only the UDVA measurements are applicable at home. Furthermore, elderly patients who undergo cataract surgery may not be able to perform digital tests unsupervised in their home environment. The introduction and usage of eHealth must always be in concordance with the patient. In addition, it should be noted that remote visual acuity testing will not completely replace ophthalmologic examination at the outpatient clinic but can enhance the efficiency of cataract care. Our results suggest that the web-based tool is useful in detecting larger changes in visual acuity but is probably not sensitive enough to reliably detect subtle changes.

Based on the results of this study, the web-based tool is validated for assessment of the visual acuity in patients who underwent cataract surgery. The web-based tool showed different outcomes compared with the conventional tests for both the UDVA and the CDVA, but most of these differences were within the established clinically acceptable limit of ±0.15 logMAR. These results are sufficient to introduce the web-based tool as a reliable screening method for detecting significant deterioration or lack of improvement of visual acuity in post-cataract patients. Our results suggest that the test can function as an interim assessment during the postoperative cataract care pathway. However, patients need to have basic digital skills to perform this web-based visual acuity assessment. Future research into this digital tool with a larger study population is necessary.

**WHAT WAS KNOWN**

- The Easee test has shown promising results for refraction and visual acuity measurements among healthy volunteers and patients with keratoconus.
- This web-based tool was not tested among patients who underwent cataract surgery before.

**WHAT THIS PAPER ADDS**

- The web-based tool is validated for assessment of the visual acuity in patients who underwent cataract surgery.
- The test is useful in detecting larger changes in visual acuity but is probably not sensitive enough to detect subtle changes.
